# Exploring the mechanism of diabetic cardiomyopathy treated with Qigui Qiangxin mixture based on UPLC-Q/TOF-MS, network pharmacology and experimental validation

**DOI:** 10.1038/s41598-024-63088-7

**Published:** 2024-05-27

**Authors:** Quancheng Han, Yan Li, Yiding Yu, Huajing Yuan, Ziqi Wang, Yonghong Guo, Jingle Shi, Yitao Xue, Xiujuan Liu

**Affiliations:** 1 Shandong University of Traditional Chinese Medicine, Jinan, People’s Republic of China; 2 Cardiology Department, Affiliated Hospital of Shandong University of Traditional Chinese Medicine, Jing Shi Road, Lixia District, Jinan, People’s Republic of China

**Keywords:** Qigui Qiangxin mixture, Diabetic cardiomyopathy, UPLC-Q/TOF-MS, Apoptosis, PI3K/AKT signaling pathway, BCL2 protein family, Cardiology, Endocrinology, Molecular medicine

## Abstract

Despite its effectiveness in treating diabetic cardiomyopathy (DCM), Qigui Qiangxin Mixture (QGQXM) remains unclear in terms of its active ingredients and specific mechanism of action. The purpose of this study was to explore the active ingredients and mechanism of action of QGQXM in the treatment of DCM through the comprehensive strategy of serum pharmacology, network pharmacology and combined with experimental validation. The active ingredients of QGQXM were analyzed using Ultra-performance liquid chromatography coupled with quadrupole time of flight mass spectrometry (UPLC-Q/TOF-MS). Network pharmacology was utilized to elucidate the mechanism of action of QGQXM for the treatment of DCM. Finally, in vivo validation was performed by intraperitoneal injection of STZ combined with high-fat feeding-induced DCM rat model. A total of 25 active compounds were identified in the drug-containing serum of rats, corresponding to 121 DCM-associated targets. GAPDH, TNF, AKT1, PPARG, EGFR, CASP3, and HIF1 were considered as the core therapeutic targets. Enrichment analysis showed that QGQXM mainly treats DCM by regulating PI3K-AKT, MAPK, mTOR, Insulin, Insulin resistance, and Apoptosis signaling pathways. Animal experiments showed that QGQXM improved cardiac function, attenuated the degree of cardiomyocyte injury and fibrosis, and inhibited apoptosis in DCM rats. Meanwhile, QGQXM also activated the PI3K/AKT signaling pathway, up-regulated Bcl-2, and down-regulated Caspase9, which may be an intrinsic mechanism for its anti-apoptotic effect. This study preliminarily elucidated the mechanism of QGQXM in the treatment of DCM and provided candidate compounds for the development of new drugs for DCM.

## Introduction

Diabetic cardiomyopathy, a disease characterized by structural and functional cardiac dysfunction in the absence of risk factors for cardiac disease (e.g., coronary heart disease, hypertension, and heart valve disease) in diabetic patients, is a severe complication of diabetes mellitus, and has emerged as a leading cause of heart failure in diabetic patients^1,2^. Some clinical trials have shown that the prevalence of heart failure among diabetic patients ranges from 19 to 26%^3–5^. The 10th edition of the IDF Diabetes Map also states that the global prevalence of diabetes will be 540 million in 2021 and is expected to rise by 12.2% to 780 million by 2045^6^. This means that a much larger population will suffer from diabetic cardiomyopathy, creating a heavy burden on global health. The pathogenesis of DCM is complex, with metabolic disorders, apoptosis, oxidative stress, and inflammation playing essential roles in the pathophysiology of DCM^7–10^, under the effect of the above mechanisms, cardiomyocyte fibrosis, cardiac diastolic dysfunction, and eventually systolic heart failure. Recent studies have shown that controlling blood glucose levels seem to be insufficient to reduce the incidence of heart failure in diabetic patients^11^; anti-heart failure medications such as ACEI/ARNI, βB, MRA, and SGLT2i have improved clinical symptoms to some extent, but hospitalization and mortality rates are still increasing yearly^12^. Therefore it is particularly crucial to seek safe and effective treatments.

According to the theory of traditional Chinese medicine, this disease belongs to the category of “Xiaoke”, “Xinji”, “Xiongbi”. Its pathogenesis is the disease for a long time, loss of qi and blood, heart and kidney damage, and chronic disease into the collaterals, heart and pulse blockage, resulting in heart displacement, development of the disease. Therefore, the treatment is mainly based on supplementing qi and nourishing blood, regulating heart and kidney, and promoting blood and collaterals. QGQXM is composed of the dried roots of *Astragalus membranaceus* Fisch. Bunge (Huangqi), the dried roots of *Angelica sinensis* Oliv. Diels (Danggui), the dried roots of *Salvia miltiorrhiza* Bunge (Danshen), the dried roots of *Radix chuanxiong* (Chuanxiong), the dried roots of *Pueraria lobata* Willd. (Gegen), the dried mature fruit pulp of *Cornus officinalis* Sieb. et Zucc. (Shanzhuyu), the dried rhizomes of *Rhodiola rosea* L. (Hongjingtian), and the dried leaves of *Epimedium sagittatum* Sieb. et Zucc. Maxim (Yinyanghuo).It is derived from the classic formula Danggui Buxue decoction and Tongmai Powder. (Chinese Guide for Diagnosis and Treatment of Diabetic Cardiomyopathy (2021–12–31)) pointed out that the ingredients of the prescription have the functions of warming “Yang” and invigorating “Qi”, promoting blood circulation and channelling pulse, and regulating heart and kidney^13^. Among them, Huangqi and Danggui supplementing “Qi” and nourishing blood are the main drugs, which can improve the complications of diabetes by affecting insulin resistance, chronic inflammation and lipid accumulation^14^. Danshen, Danggui, Chanxiong and Gegen promote blood circulation and reduce the risk of bleeding while preventing blood clotting^15^. Yinyanghuo, Hongjingtian and Shanzhuyu can improve mitochondrial function, regulate autophagy, inhibit the expression of Col IV, FN and IL-6, and play a role in inhibiting diabetic complications and protecting the heart^16–18^. Although there are many related studies, the specific mechanism of its treatment of diabetic cardiomyopathy is still not fully understood. Chinese medicines are characterized by multi-component and multi-target. Serum pharmacology suggests that the components of Chinese medicines that can be absorbed into the bloodstream are the active components that exert their medicinal effects. For this reason, we combined UPLC-Q/TOF-MS with network pharmacology to study the interactions between TCM components and disease networks. UPLC-Q/TOF-MS is the method of choice for the identification of drugs and metabolites in biological fluids, and it has the advantages of being accurate, rapid, and comprehensive, which makes it an essential tool for the identification of the components of TCM^19,20^. Network pharmacology is an emerging interdisciplinary discipline that integrates biology, informatics, artificial intelligence, and other disciplines to elucidate the targets and mechanisms of action of compounds at the molecular level by establishing network relationships between compounds and biological information^21^.

In this study, UPLC-Q/TOF-MS was used to analyze the active components in rat drug-containing serum, to elucidate the mechanism of action of QGQXM for the treatment of DCM using network pharmacology, and to verify the core mechanism through animal experiments. The study process is shown in graphical abstract.

## Materials and methods

### Animals

Fifty Wistar male rats (8 weeks, 200 ± 20 g) were purchased from Beijing Vital River Laboratory Animal Science and Technology Co., Ltd. and clean-fed in the Animal Experimentation Center of the Affiliated Hospital of Shandong University of Traditional Chinese Medicine, where humidity was maintained at 50–60%; temperature was held at 20–25 °C, and regular feed was fed with free water. The experimental procedures were approved by the Ethics Committee for Animal Experiments of Shandong University of Traditional Chinese Medicine and were performed in accordance with the standards set forth in the Guidelines for Animal Experiments of the Chinese Medical Ethics Committee (2021-007).

### UPLC-Q/TOF-MS analysis

#### Preparation of drug-containing serum

QGQXM was bought from the Affiliated Hospital of Shandong University of Traditional Chinese Medicine (Shandong, China). QGQXM composition is shown in Table 1. The dose administered to rats (based on the raw drug content) was 6.25 times higher than that allocated to humans^22^. In the present study, the human clinical dose was 135 g/d, and the adult body weight was 70 kg, while the rat dose was 135 g/70 kg × 6.25 ≈ 12 g/kg. Two groups of ten rats were divided at random into control and QGQXM groups (n = 5 in each group). The control group was given 0.9% saline two ml/d by gavage, and the QGQXM group was assigned 12 g/kg QGQXM. Seven consecutive days of gavage were administered to rats. Before sampling, all rats were fasted for 12 h and anesthetized by intraperitoneal injection of 40 mg/L sodium pentobarbital 2 h after the last gavage. It was necessary to take blood from the abdominal aorta, and the blood samples were centrifuged at 3000 r/min for 15 min after 30 min of rest, and the supernatant was collected for UPLC-Q/TOF-MS analysis. Another group of traditional Chinese medicine solution was set up as a positive control for assessing the composition of traditional Chinese medicine into blood.

**Table 1:** Composition of QGQXM.

Academic name	English name	Chinese name	Dose of crude drug (g)	Dose of granule (g)
Radix Astragali	Root of Membranous Milkvetch	HUANGQI	30	12
Radix Salviae liguliobae	Root of Ligulilobe sage	DANSHEN	20	10
Radix Angelicae sinensis	Root of Chinese Angelica	DANGGUI	20	3.4
Radix et Rhizoma Rhodiolae	All—grass of Rhodiola	HONGJINGTIAN	20	2
Fructus Corni	Asiatic Cornelian Cherry Fruit	SHANZHUYU	12	1.7
Epimedium	Epimedium	YINYANGHUO	12	0.6
Radix Puerariae	Root of lobed kudzuvine	GEGEN	12	4.8
Rhizoma Chuanxiong	Szechuan lovage root	CHUANXIONG	9	4.5

#### UPLC-Q/TOF-MS data analysis

The data were collected and processed using SCIEX OS software, using multiple confidence criteria. These criteria included mass accuracy, retention time, isotopes, and compound library matches. In this experiment, the screening of the target in the absence of standards can be accomplished by searching the TCM MS/MS Library (which contains secondary data of more than 1000 TCM compounds), a secondary database of traditional Chinese medicines configured by SCIEX OS, based on the compound's first-order accurate mass number, isotope distribution ratio, and MS/MS. Specific procedures and chromatographic and mass spectrometric conditions are described in Supplementary Material 1.

### Data collection

Chinese medicines are characterized by multi-components, and according to the theory of serum pharmacology, the components of a drug that are absorbed into the bloodstream are its active ingredients that exert biological effects. Therefore, we set the blank serum as the negative control group and the herbal solution as the positive control group. The screening criteria for the prototypical components of the blood entry are as follows: firstly, the overlapping components in the drug-containing serum and the herbal solution, and secondly, element does not exist in the blank serum. To predict the potential targets of QGQXM blood-entry components, We obtained SMILES using the PubChem database (https://pubchem.ncbi.nlm.nih.gov/) from the UPLC-Q/TOF-MS results. SMILES was entered into SwissTargetPrediction (https://www.swisstargetprediction.ch/), Homo sapiens was selected for the species setting, and compound targets with zero probability were excluded to obtain potential therapeutic targets for QGQXM. The keyword "Diabetic Cardiomyopathy" was used to search the human Mendelian inheritance database OMIM (http://www.omim.org/), GeneCards database (https://www.genecards.or/), select Homo sapiens for the species setting, and the acquired genes were merged and de-emphasized to obtain the disease targets of DCM. The blood component targets and DCM disease targets were entered into the Venny 2.1.0 (https://bioinfogp.cnb.csic.es/tools/venny/index.html) online platform to receive the intersection, i.e., the potential target of action of QGQXM for the treatment of DCM.

### Protein–protein interaction (PPI) network

The targets of QGQXM for the treatment of DCM were uploaded to the STRING database (https://cn.string-db.org/), and the PPI (protein–protein interactions) network of potential targets of QGQXM compounds for the treatment of DCM was analyzed. Homo sapiens was selected for the biological column, and the required minimum interaction score for the network was 0.7. The results were then imported into the Cytoscape (Version 3.9.1) software for the protein–protein interaction (PPI) network. The minimum interaction score for the network was 0.7. The results were then imported into Cytoscape (Version 3.9.1) software for protein–protein interaction visualization analysis and screening of core targets based on the degree value.

### GO function and KEGG pathway enrichment analysis

Using R (4.2.1), GO and KEGG enrichment analysis was performed with the ClusterProfiler package after ID conversion of the input molecule lists. Homo sapiens was selected as the species, and the ID conversion library (package): org.Hs.eg.db^23^. *p* < 0.05 indicated that the enrichment was statistically significant.

### Animal model and treatment

Forty Wistar male rats (8 weeks, 200 ± 20 g) were randomly divided into four groups (n = 10 in each group): control (CON), DCM model (MOD), QGQXM treatment (QGQXM) , and metformin (MET). After one week of acclimatization feeding with regular feed, the CON group continued to be fed standard feed; the MOD, QGQXM and MET groups were given high-fat feed for four weeks, and then intraperitoneally injected with 35 mg/kg streptozotocin (STZ) to continue the high-fat feeding to induce diabetes mellitus modeling^24^. After four weeks, blood was taken via the caudal vein, and fasting glucose was detected to be greater than 16.7 mmol/L. At the same time, a cardiac ultrasonography was used to measure left ventricular ejection fraction and short-axis systolic rate to validate the model. After successful modeling, the CON and MOD groups were assigned two mL/d of saline by gavage, the QGQXM group was given 12 g/kg/d of QGQXM, and the MET group was assigned 89 mg/kg/d of metformin (the clinical dose in humans is 1000 mg/d, and the body weight of an adult is 70 kg, while the dose in rats is 1000 mg/70 kg × 6.25 ≈ 89 mg/kg). The intervention was performed for eight weeks.

### Echocardiographic assessment

After eight weeks of treatment, rats were anesthetized with pentobarbital and underwent transthoracic echocardiographic ultrasonography. Two-dimensional guidance of LV internal diameter was obtained from the short-axis view at the muscle level, and left ventricular ejection fraction (LVEF) and left ventricular short-axis systolic rate (LVFS) were measured in M-mode. The above data were measured at least three times and averaged.

### Hematoxylin–eosin staining and Masson staining

As an alkaline stain, hematoxylin can stain chromatin and nucleic acids to blue-violet colors in the nucleus and cytoplasm. Red cytoplasm and extracellular matrix are stained with eosin stain, an acidic stain. The morphology of tissue cells can be observed by hematoxylin–eosin staining. Masson staining can selectively display collagen fibers and myofibers, which is an essential test for assessing tissue fibrosis. To follow the myocardial injury and the degree of fibrosis in DCM rats, we isolated heart tissue from rats and fixed it with 4% paraformaldehyde. The tissues were dehydrated, embedded in paraffin and cut into 4-μm slices, stained with hematoxylin–eosin and sealed. Light microscopic observation of pathological changes in cardiomyocytes. The paraffin-embedded cardiac tissues of rats were sectioned again, stained with Masson staining, and subjected to quantitative analysis of fibrosis using Aipathwell software (ServiceBio, Wuhan, Hubei, China) to assess the extent of myocardial fibrosis. The detailed algorithm is described in Supplementary Material 2.

### TUNEL staining

TUNEL staining detects the breakage of nuclear DNA during apoptosis and is used to calculate the apoptosis rate of cells. After TUNEL staining of tissue sections, positive area analysis was performed using Aipathwell software (ServiceBio, Wuhan, Hubei, China), and the detailed algorithm is described in Supplementary Material 3.

### Western blotting

The cardiac tissue was minced and rinsed in ice-cold PBS to remove blood and debris. Samples were then placed into centrifuge tubes containing tissue RIPA buffer (Servicebio, G2002) and PMSF (Beyotime, ST506-2), and homogenized evenly using a mechanical tissue homogenizer. The lysate mixture was subsequently incubated on ice with shaking for a brief period to facilitate cell membrane rupture and protein release. The protein concentration was determined using the BCA Protein Assay Kit (Solarbio, PC0020). Subsequently, the protein samples were mixed with the loading buffer to equal concentrations, and the mixture was heated at 100 °C for 5 min to denature the proteins. Samples were loaded onto SDS-PAGE gels (Servicebio, G2037) according to protein concentration and electrophoresed, then transferred onto PVDF membranes (BIO-RAD, 1620177). The PVDF membrane was washed three times with TBST. Subsequently, the PVDF membrane was immersed in 5% skim milk (BD, 232100) and incubated on a shaker at room temperature for two hours. After incubation, the membrane was washed three times with TBST for 10 min each. The corresponding concentrations of primary antibodies were added: Akt (pan) (C67E7) Rabbit mAb (1:1000, CST, 4691), Phospho-Akt (Ser473) (D9E) XP (1:2000, CST, 4060), PI3 Kinase p85 (19H8) Rabbit mAb (1:1000, CST, 4257), Phospho-PI3 Kinase p85 (Tyr458)/p55 (Tyr199) Antibody # (1:2000, CST, 4228), Bcl-2 (2:1000, Abcam, ab182858), Caspase 9 (1:1000, proteintech, 10380-1-AP) and incubated overnight. After another three washes with TBST, the membrane was incubated with the appropriate concentration of horseradish peroxidase-conjugated secondary antibody (CST, #7074) solution for two hours. Following re-washing with TBST, the membrane was developed using ECL Substrate A (Vazyme, E412-02) and Peroxide Solution B (Vazyme, E412-02). The prepared solution was evenly spread onto the PVDF membrane, incubated for 3 min in the dark, and then imaged. Image J software was used to analyze the grayscale values of each band. The relative expression levels were calculated based on the grayscale ratio between the target protein and the internal reference protein bands.

### Statistical analysis

In this study, data were analyzed using SPSS 27.0.1 (IBM Corp, USA), and are presented as means and standard deviations. The difference between groups was tested using independent sample T-tests, and for data not following a normal distribution, independent sample non-parametric tests were used. A P-value of less than 0.05 was considered statistically significant. Data visualization was performed using GraphPad Prism 8.0.

### Ethical approval and consent to participate

All trial procedures were approved by the Ethics Committee of Shandong University of Traditional Chinese Medicine (2021-007). All authors have read this manuscript and agree to its publication.

### Statement

Confirmation of this study is according to ARRIVE guide (https://arriveguidelines.org). No human samples were involved in this study. The experimental procedures were approved by the Ethics Committee for Animal Experiments of Shandong University of Traditional Chinese Medicine and were performed in accordance with the standards set forth in the Guidelines for Animal Experiments of the Chinese Medical Ethics Committee (2021-007).

## Results

### 25 Blood-entering components were identified by UPLC-Q/TOF-MS

**Figure 1:**
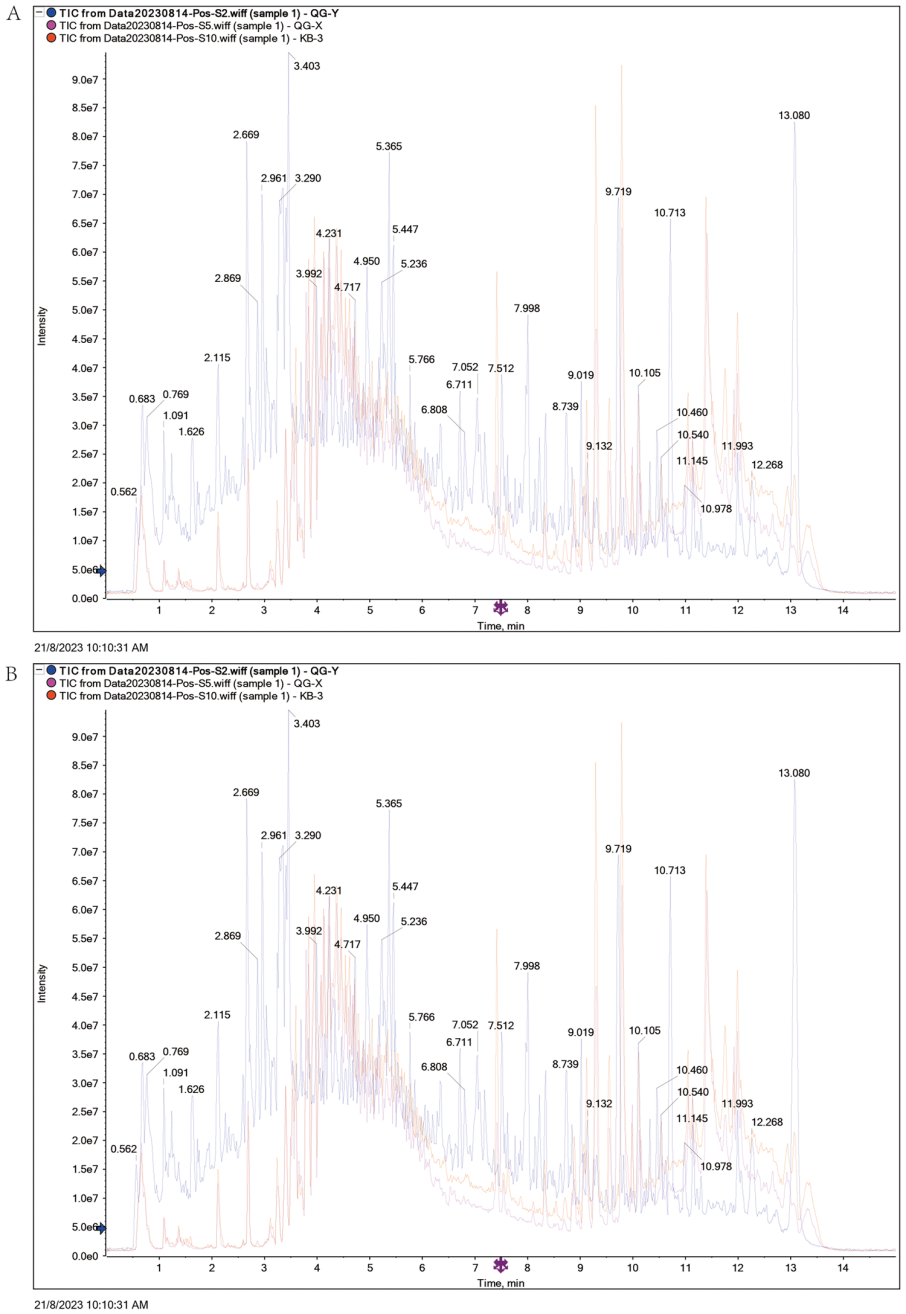
UPLC-Q/TOF-MS was used to identify QGQXM compounds in (**A**) positive ion and (**B**) negative ion mode. Interim kb represents blank serum, QG-Y represents traditional Chinese medicine solution, and QG-X represents drug-containing serum.

To identify the blood-entering components of QGQXM, we performed UPLC-Q/TOF-MS analysis on rat drug-containing serum, control serum, and Chinese medicine solution. Figure 1 illustrates that each group's total ion chromatogram behaved differently in the positive and negative ion modes, suggesting compositional differences. Utilizing the UPLC-Q/TOF-MS analysis technique and adhering to our blood-entry component screening standards, 25 prototypical components entering the bloodstream were identified, as detailed in Table 2.

**Table 2:** Identification of blood-entry components from QGQXM in serum of rats.

Rank	Name	Formula	Mass (Da)	RT (min)	Score (%)	Area
1	Limonin	C_26_H_30_O_8_	470.1941	6.92	100	877
2	Scopoletin	C_10_H_8_O_4_	192.0423	0.71	99	2106
3	Adenosine	C_10_H_13_N_5_O_4_	267.0967	1.61	99	211
4	Daidzein	C_15_H_10_O_4_	254.0579	5.22	97	589
5	Puerarin	C_21_H_20_O_9_	416.1107	3.32	96	964
6	4-Hydroxyphenylpyruvic acid	C_9_H_8_O_4_	180.0423	0.67	95	4997
7	Farrerol	C_17_H_16_O_5_	300.0998	2.74	93	2732
8	Gramine	C_11_H_14_N_2_	174.1157	0.68	92	3671
9	Shikimic acid	C_7_H_10_O_5_	174.0528	0.68	92	217
10	Suberic acid	C_8_H_14_O_4_	174.0892	0.73	92	1209
11	Allantoin	C_4_H_6_N_4_O_3_	158.0440	3.52	92	1436
12	Nicotinic acid	C_6_H_5_NO_2_	123.0320	1.38	89	484
13	Trigonelline	C_7_H_7_NO_2_	137.0477	0.71	89	2289
14	7-ethyl-10-hydroxycamptothecin	C_22_H_20_N_2_O_5_	392.1372	6.50	87	184
15	Baicalin	C_21_H_18_O_11_	446.0849	3.43	86	948
16	Paederoside	C_18_H_22_O_11_S	446.0883	3.42	86	662
17	p-Coumaric acid	C_9_H_8_O_3_	164.0473	12.48	85	1128
18	Coumestrol	C_15_H_8_O_5_	268.0372	6.71	78	566
19	Arg(Arginine)	C_6_H_14_N_4_O_2_	174.1117	0.68	75	4479
20	7-O-Methylmangiferin	C_20_H_20_O_11_	436.1006	3.36	73	764
21	Ascorbic acid	C_6_H_8_O_6_	176.0321	3.61	72	1396
22	Curcumenol	C_15_H_22_O_2_	234.1620	11.92	68	1885
23	Icarrin	C_33_H_40_O_15_	676.2367	5.44	68	2892
24	Hydroxycitric acid	C_6_H_8_O_8_	208.0219	0.21	66	396
25	2-Pyrrolidinone	C_4_H_7_NO	85.0528	3.25	63	1806

### Target acquisition

Using the PubChem database and Swiss Target Prediction database, target prediction was performed on the 26 components, and after removing duplicates and targets with a confidence level of 0, a total of 426 potential targets were acquired. A total of 2389 DCM-related targets were obtained from the genecards database and OMIM database. Taking the intersection of the predicted targets of the incoming components with the disease targets of DCM, a total of 121 common targets were obtained as potential targets of QGQXM for treating DCM.

### PPI network construction and analysis

**Figure 2:**
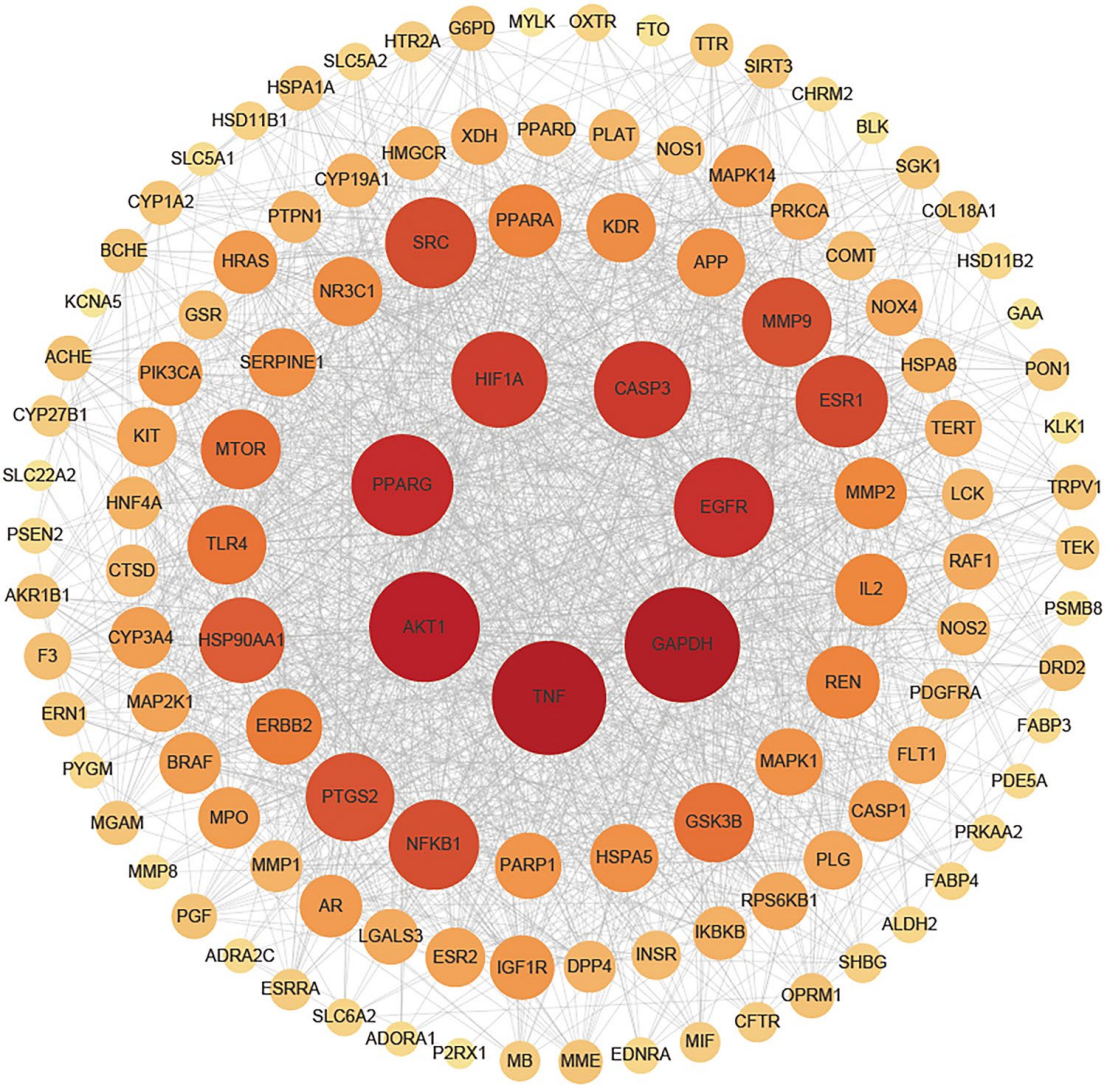
QGQXM's core target proteins were predicted using PPI networks using common targets, and target genes were arranged into four concentric circles based on node degree. There is a range of 71–91 node degrees for the innermost target gene, the second layer is between 40 and 67, the third layer is between 22 and 38, and the outermost layer is between 1 and 20. There is an association between the Degree parameter and the size and color of the corresponding node in the figure.

The PPI network was constructed using the STRING database and visualized and analyzed by Cytoscape 3.9.1, see Fig. 2. There are 121 nodes and 1699 edges in the network. The results showed that the top-ranked points in terms of degree value were GAPDH (degree = 91), TNF (degree = 90), AKT1 (degree = 86), PPARG (degree = 77), EGFR (degree = 75), CASP3 (degree = 72), HIF1A (degree = 71), ESR1 (degree = 67), SRC (degree = 66), NFKB1 ( degree = 65) can be inferred as the core target of action of QGQXM for DCM treatment.

### GO function and KEGG pathway enrichment analysis

**Figure 3:**
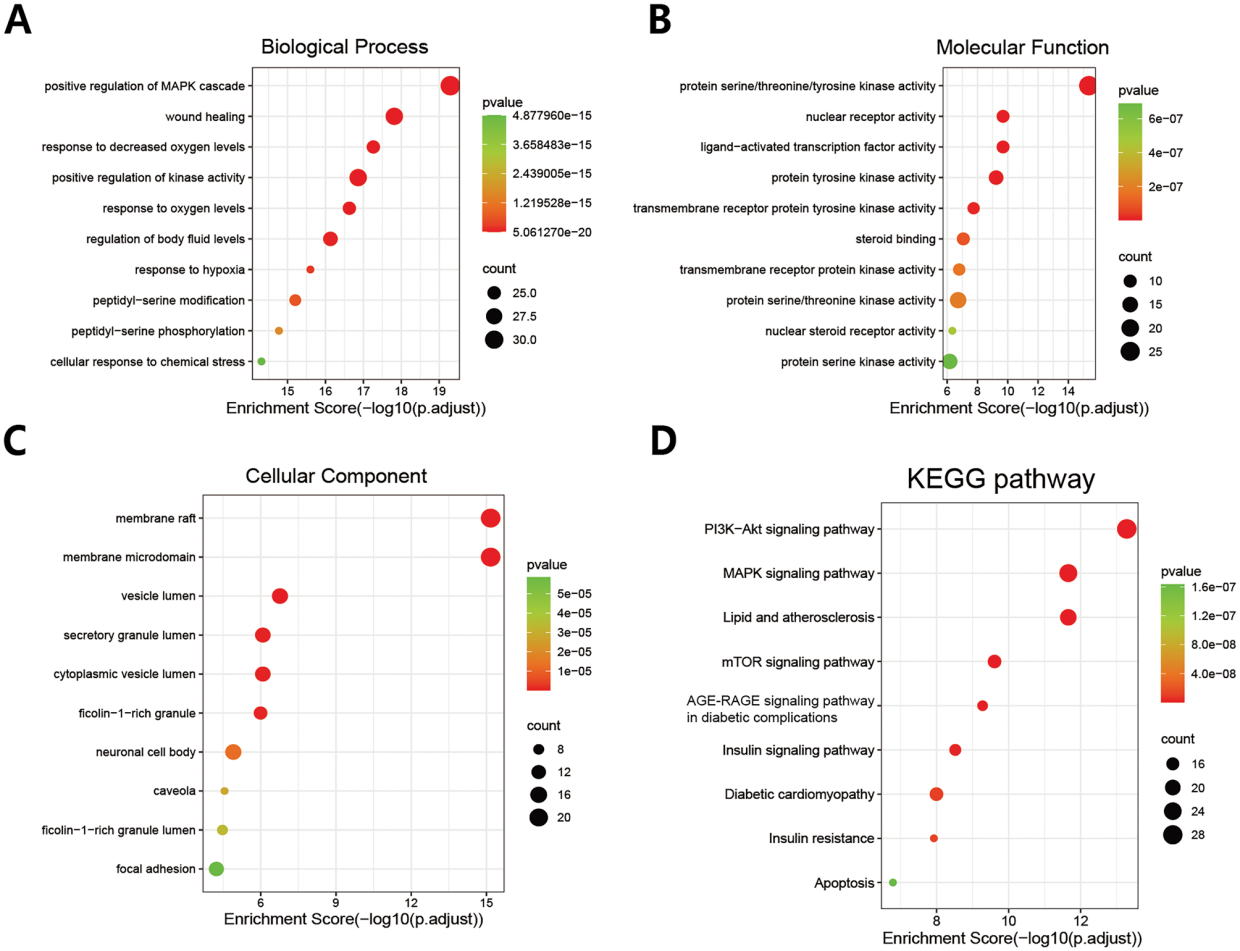
GO and KEGG enrichment analysis identified the pathways associated with QGQXM treatment of DCM. (**A**) GO enrichment analysis of (**A**) biological processes, (**B**) molecular functions and (**C**) cell localization of the top ten enrichment items. (**D**) The top ten DCM related pathways in KEGG pathway analysis.

**Figure 4:**
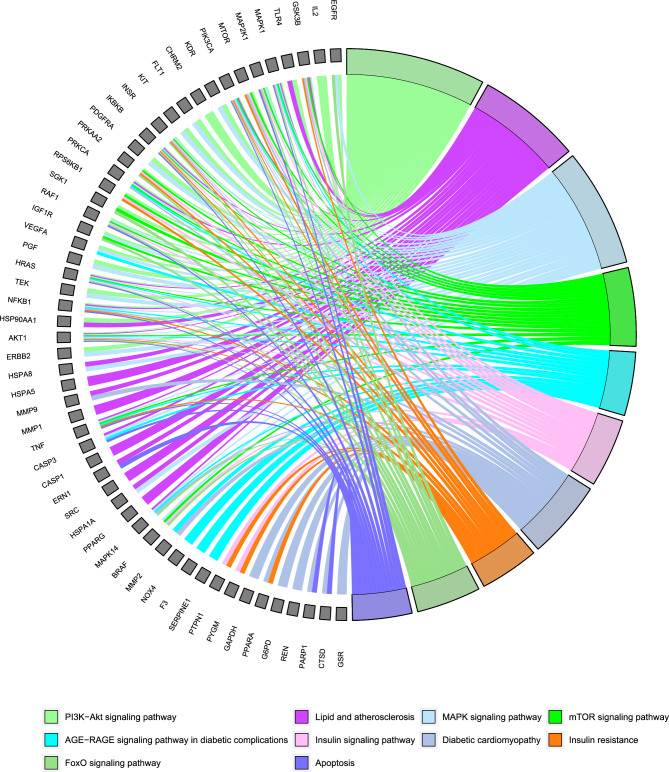
KEGG path enrichment analysis of the relationship between the path and the target, gray nodes represent the target, color nodes represent the path, line represents the relationship between the path and the target.

To explore the biological functions and signaling pathways of QFQXM targets for DCM, we performed GO function and KEGG pathway enrichment analysis on the target genes using R (4.2.1) We selected the Benjamini-corrected *P* < 0.01 as the significance threshold. The enrichment results were also visualized using R. The GO function analysis showed 2121 biological processes (BPs), of which 1302 were significantly related to protein phosphorylation, protein kinase activation, signal transduction, oxidation level regulation, and damage repair, etc. Based on the GO function analysis, 2121 biological processes (BP) were identified, of which 1302 were significantly related to protein phosphorylation, protein kinase activation, signal transduction, oxidation level regulation, and damage repair. Molecular Function (MF) totaled 148 items, of which 65 were significantly related, mainly in protein kinase activity, transcriptional activity, protein binding, hormone binding, and binding of related transcription factors. A total of 88 entries were analyzed for cellular localization (CC), and 45 entries were significantly enriched, mainly in the structures of lipid membrane, cytosol, and cytoplasm, and to a lesser extent in some of the structures of the nuclear membrane. The top 10 entries of BP, CC, and MF were presented in the form of bubble plots (Fig. 3). A total of 161 pathways were obtained from the analysis of KEGG pathways, and 132 (*P* < 0.01) entries were significantly correlated, among which the PI3K- Akt, MAPK, mTOR, Insulin, Insulin resistance, and Apoptosis signaling pathway are considered to be the core pathways of QGQXM for DCM treatment. Figure 3 shows the results of 10 relevant pathways. To clarify the interrelationship between core targets and signaling pathways, we used R software to perform visual analysis. Figure 4 demonstrates the interaction between signaling pathways and core targets. Based on the results of the KEGG enrichment analysis, we chose the PI3K-Akt signaling pathway, ranked the highest among the pathways closely related to DCM, as the core pathway for validation.

### QGQXM can improve cardiac function in DCM rats

**Figure 5:**
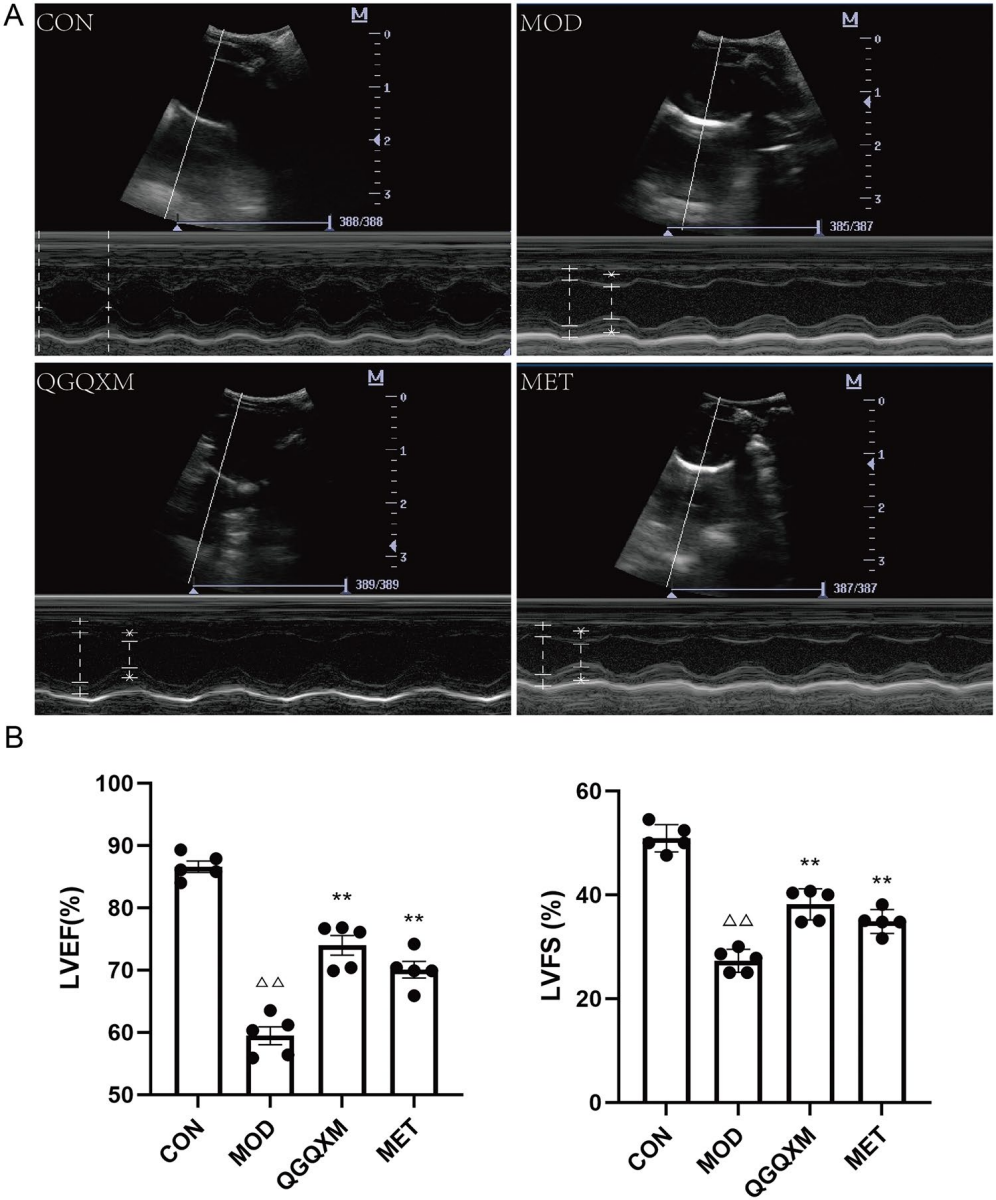
QGQXM can increase LVEF and LVFS in DCM rats (n = 5). (**A**) Representative M-type echocardiography of rats. (**B**) Summary of left ventricular ejection fraction (LVEF) in each group. (**C**) Left ventricular short axis shrinkage (LVFS) of each group was summarized, ΔΔ compared with the control group, *P* < 0.01, ΔΔΔ compared with the control group, *P* < 0.001; ** compared with the model group, *P* < 0.01.

Systolic heart failure is the ultimate manifestation of DCM. In order to evaluate the effect of QGQXM on cardiac function in DCM rats, we performed echocardiography on the treated rats. In Fig. 5, the results showed that ventricular systolic and diastolic functions were reduced in the model group, and LVEF and FS were significantly reduced (*P* < 0.001). Compared with the model group, LVEF and FS were elevated in both the QGQXM and MET groups of rats (*P* < 0.01). These data proved that QGQXM could effectively improve the cardiac function of DCM rats and had a better anti-DCM effect.

### QGQXM can alleviate the severity of myocardial injury in DCM rats

**Figure 6:**
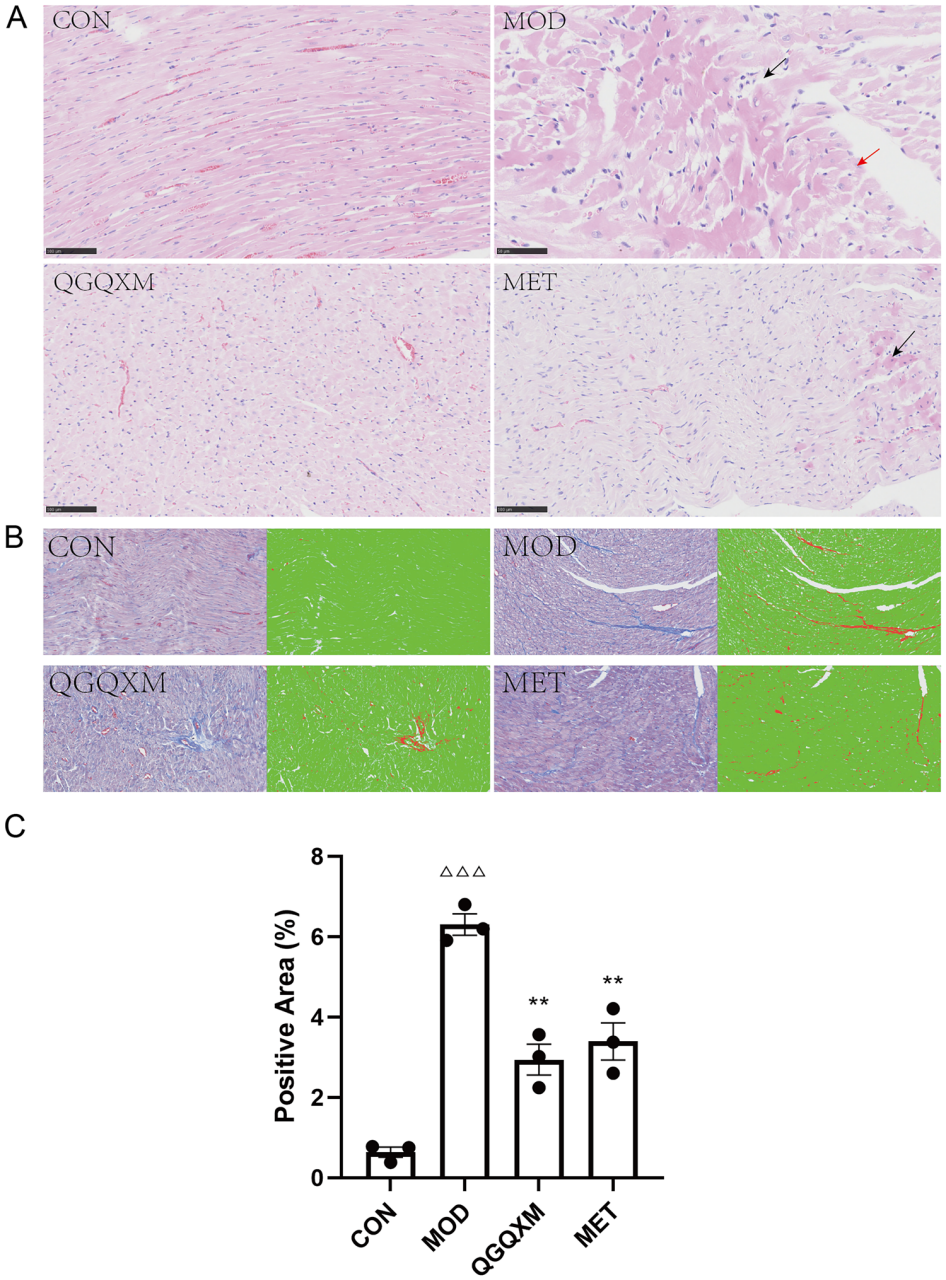
QGQXM can improve myocardial cell damage and reduce fibrotic area. (**A**) HE staining of representative images. The black arrows represent necrotic cardiomyocytes and the red arrows represent cardiomyocyte watery lesions. (**B**) Mosson dyeing representative images. (**C**) Summary of Mosson staining results in each group (n = 3); ΔΔΔ Compared with the control group *P* < 0.001; **P < 0.01 compared with model group.

Cardiomyocyte damage and fibrosis are pathological features of DCM. Therefore, we performed H&E staining and Masson staining on the myocardial tissues of DCM rats to observe the damage of the myocardium and the degree of fibrosis. As shown in Fig. 6A, in H&E staining, the cells in the control group were normal in morphology, neatly arranged, clearly demarcated, with a consistent direction, and the transverse lines of the cardiomyocytes were clear, bright, and dark. Compared with the standard group, necrosis of cardiomyocytes (black arrowheads), eosinophilic homogenous cytoplasm, cytosolic solidification, and irregular shape could be seen in the model group; a small number of cardiomyocytes with hydropic degeneration (red arrowheads), cell swelling, and sparse and light-stained cytoplasm could be seen in the model group. Compared with the model group, the morphology of QGQXM cardiomyocytes was more regular, with uniform coloring of myocardial fibers, clear cell differentiation, consistent direction, clear transverse stripe of cardiomyocytes, and light and dark intervals. In the MET group, occasional pitting necrosis of cardiomyocytes was seen (black arrows), with a small amount of cytosolic solidification, and irregular shape; the rest of the cardiomyocardial fibers was uniformly colored, cell distinction was clear, and the direction was consistent, and the transverse stripe of cardiomyocytes was clear, and light and dark intervals were observed. The rest of myocardial fibers were uniformly colored, with clear cell demarcation and consistent direction, and the transverse lines of myocytes were clear, bright and dark. In Fig. 6B and C, Masson staining shows, the fibrotic area was less in the CON group and significantly more in the MOD group, and the fibrotic area was significantly lessen in the QGQXM and MET groups compared with the MOD group, *P* < 0.05. In summary, QGQXM could reduce the degree of cardiomyocyte damage, decrease the fibrotic area of the myocardium, and play the role of anti-DCM.

### QGQXM reduced the apoptosis rate of myocardial cells in DCM rats

**Figure 7:**
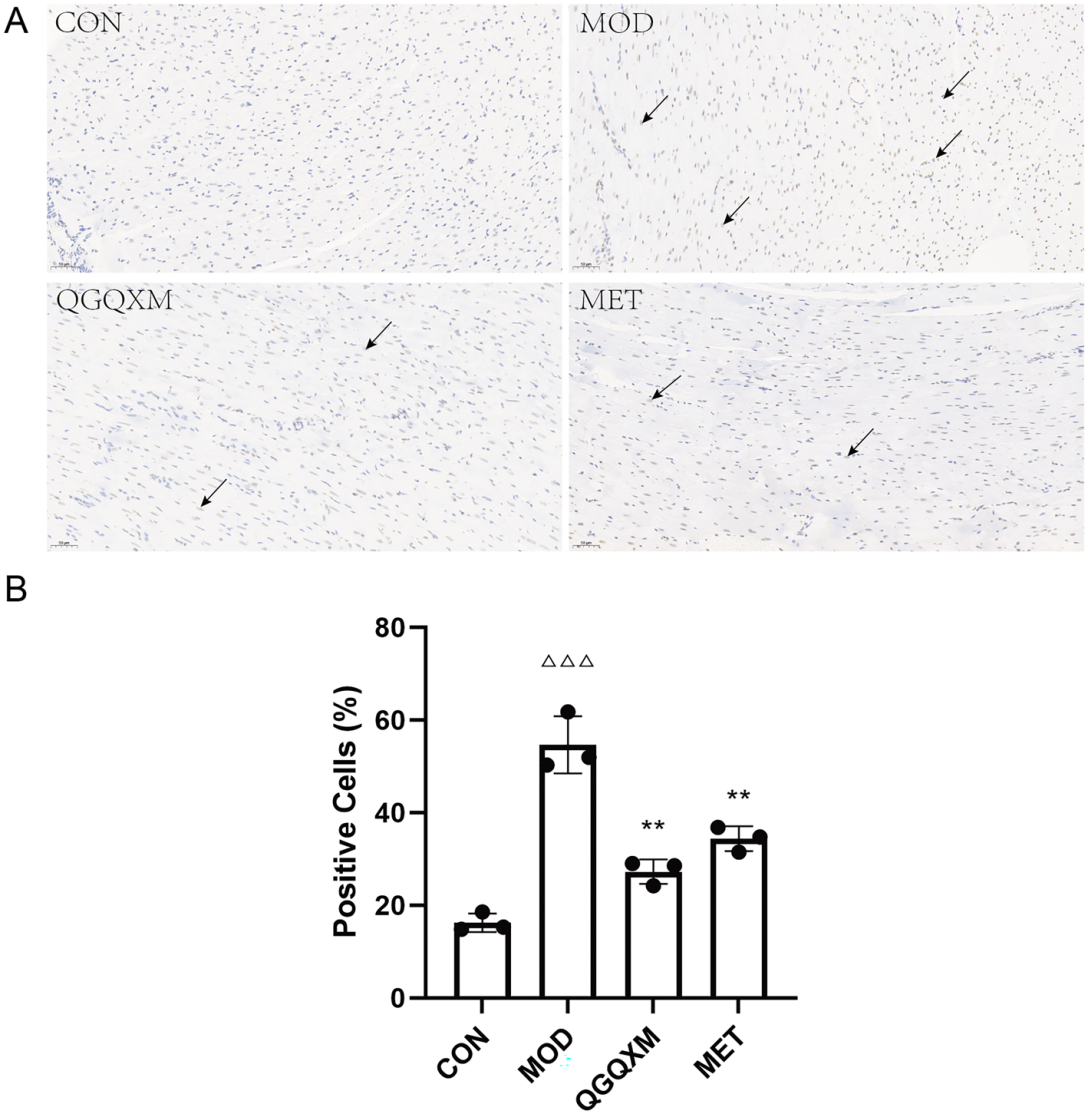
QGQXM can improve the apoptosis rate of cardiomyocytes. (**A**) TUNEL staining representative images of each group. The black arrows show TUNEL positive cells (apoptotic cells). (**B**) Summary of TUNEL positive rate in each group (n = 3), ΔΔΔ compared with the control group, *P* < 0.001; **P < 0.01 compared with model group.

Apoptosis induced by the PI3K-AKT signaling pathway plays an essential role in the pathogenesis of DCM^25^, so to assess the apoptosis of rat cardiomyocytes, we performed TUNEL staining on tissue sections. As shown in Fig. 7, only a small number of TUNEL-positive cells were found in the myocardial tissues of the CON group; TUNEL-positive cells were significantly increased in the MOD group compared with the CON group, *P* < 0.001; and positive cells were significantly reduced in the QGQXM group and the MET group compared with the model group, *P* < 0.01. The TUNEL results indicated that QGQXM could effectively improve the apoptosis in the cardiac tissues of the rats with DCM, thereby exerting a protective effect on the myocardium.

### QGQXM can modulate the protein expression in the PI3K-AKT signaling pathway

**Figure 8:**
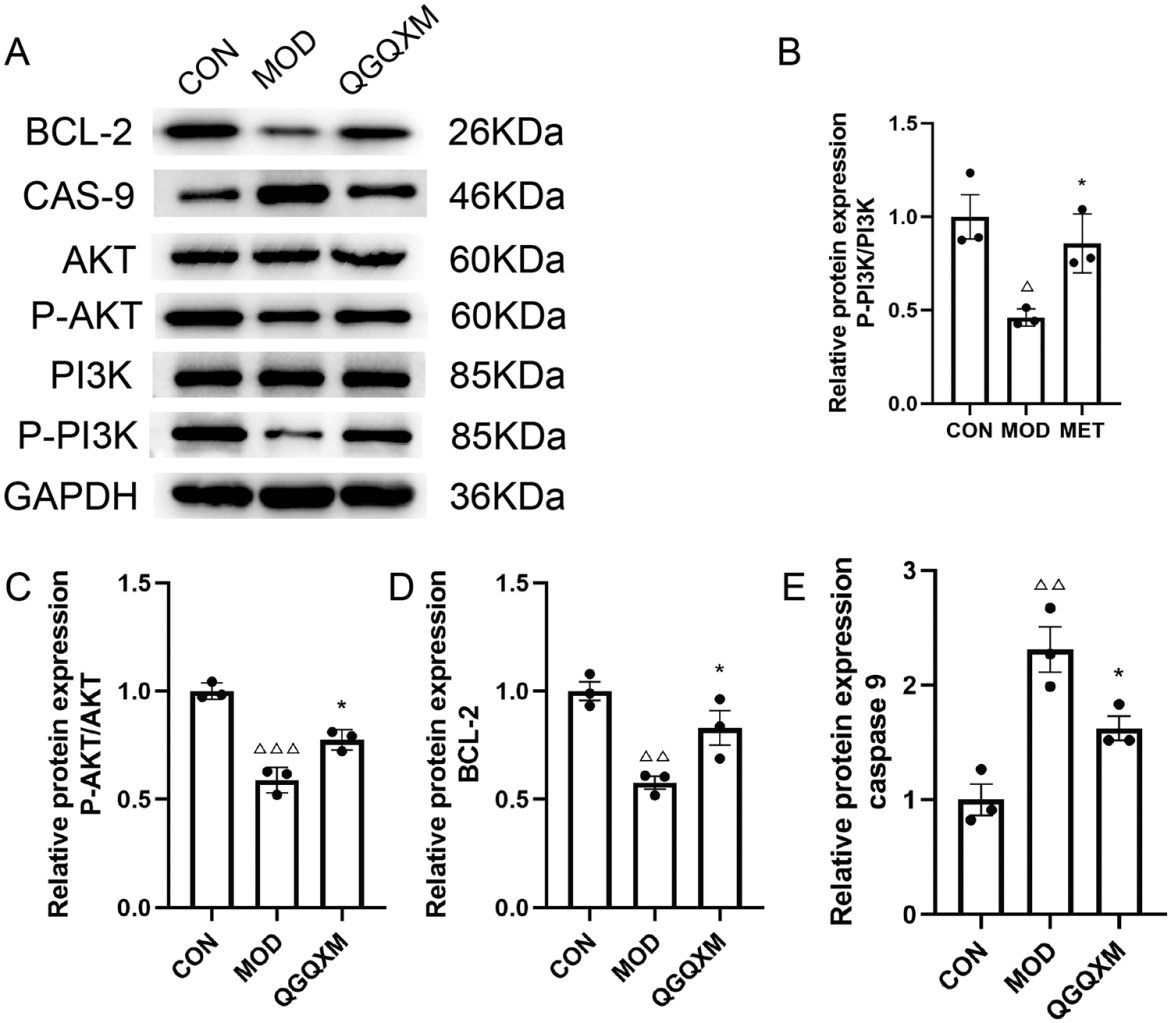
(**A**) Representative images of the strips by Western blot. (**B**) phosphorylated PI3K protein level (P-PI3K); (**C**) phosphorylated AKT protein level (P-AKT); (**D**) content of Bcl-2, (**E**) content of caspase9. The relevant expressions were summarized (n = 3), ΔΔ compared with the control group *P* < 0.01, ΔΔΔ compared with the control group *P* < 0.001, * compared with the model group *P* < 0.05.

To verify the regulatory effect of QGQXM on the PI3K-AKT signaling pathway and its induced apoptosis-related molecules, Western blot analysis revealed the protein levels for downstream apoptosis factors Bcl-2 and caspase-9 along with PI3K-AKT signaling pathways (Fig. 8). The results showed that P-PI3K, P-AKT, and Bcl-2 were significantly decreased (*P* < 0.001), and caspase-9 expression was significantly elevated (*P* < 0.001) in the model group compared with the control group. Compared with the DCM group, the expression levels of P-PI3K, P-AKT, and Bcl-2 were significantly elevated after QGQXM treatment (*P* < 0.01), with the most significant difference in P-PI3K (*P* < 0.001), and the expression of the pro-apoptotic factor caspase-9 was down-regulated (*P* < 0.01). These results indicated that QGQXM activated the PI3K-AKT signaling pathway up-regulated the apoptosis protective factor Bcl-2, and inhibition of apoptosis-inducing factor caspase9 expression, which exerted myocardial protection by inhibiting apoptosis. WB raw data are available in Supplement 4.

## Discussion

The morbidity and mortality of diabetic cardiomyopathy are high worldwide. Although new therapeutic methods are constantly being updated, they still cannot effectively stop the development of the disease. Traditional Chinese medicine (TCM) has the advantages of multi-component and multi-targets, which can comprehensively regulate multiple mechanisms of DCM occurrence and development, fully reflecting the holistic concept of TCM. In this study, we systematically and thoroughly analyzed the blood-entry components of QGQXM and its mechanism of treating DCM by combining serum pharmacology, serum medicinal chemistry, and network pharmacology. We validated them in vivo by constructing a rat model of DCM. It provides the theoretical basis and data support for QGQXM to treat DCM. Researchers found that QGQXM could improve the cardiac function of DCM rats, improve the degree of cardiomyocyte injury and fibrosis, inhibit apoptosis by regulating the PI3K-Akt signaling pathway, and play a protective role for the myocardium of DCM rats.

Ultra performance liquid chromatography mass spectrometry (UPLC-Q/TOF-MS) analysis can accurately identify the chemical constituents in TCM and serum, and the results are more objective and accurate compared with the TCM constituents database, providing scientific data support for mechanism analysis. The mass spectrometry results showed that 25 prototypical components were introduced into the blood, mainly azurin, soybean flavonoids, epimedium glycosides, baicalin, scopolamine, etc. The results of mass spectrometry showed 25 prototypical components in the blood. These components acted on different mechanisms to exert anti-DCM effects. For example, azurin inhibits glutamate-induced reactive oxygen species expression, apoptosis, and attenuates mitochondrial damage in HT3 cells^26^. Soy flavonoids can improve hyperglycemia, insulin resistance, and inflammation, and reduce necrosis and fibrosis of cardiac tissues^27,28^. Icariin inhibits cardiomyocyte apoptosis and cardiac remodeling^29,30^. Baicalin prevents insulin resistance, metabolic dysfunction, cardiac fibrosis, and heart failure^31,32^. Scopolamine improves insulin resistance and promotes insulin secretion^33^. Accordingly, we conducted further studies to explore the potential targets and pathways of these components for the treatment of DCM through network pharmacology to provide scientific data to support our further research.

Enrichment analysis showed that there are a large number of signaling pathways that may be involved in QGQXM for the treatment of DCM. The PI3K-Akt signaling pathway is not only the topmost among the pathways related to DCM, but also the key targets in the pathway, such as AKT1 and PIK3CA, are also the core targets in the PPI network. Therefore, we took the PI3K-Akt signaling pathway as a target for validation. The PI3K-Akt signaling pathway can regulate various cellular processes, which are closely related to cell proliferation, growth, metastasis, and apoptosis^33^. The PI3K-Akt signaling pathway has been found to play a cardioprotective role in cardiomyocytes by inhibiting apoptosis^34,35^. PI3K, fully known as phosphatidylinositol 3-kinase, has three different types^36^, of which only class I can act as lipid phosphorylator upon upstream signaling stimulation^37^. PI3K (class I) is a heterodimer consisting of the catalytic subunit P110 and regulatory subunit P85^38^. Activation of PI3K is initiated by binding extracellular growth factors to transmembrane receptor tyrosine kinases (RTKs). When the ligand binds to the receptor, the RTK is activated and recruits PI3K at the lipid membrane. The catalytic subunit P85 binds to the RTK, releasing the catalytic subunit P110, which completes the phosphorylation of PI3K^39^. Activated PI3K anchors to the lipid membrane and catalyzes the phosphorylation of Phosphatidylinositol 4,5-bisphosphate (PIP2) to Phosphatidylinositol 3,4,5-triphosphate (PIP3). PIP3 recruits AKT, identical to mTOR complex PIP3 recruits AKT and, together with mTOR complex 2 (mTORC2), fully activates AKT^40^. Once phosphorylated, AKT detaches from the lipid membrane, migrates to the cytoplasm and nucleus, and binds to downstream target proteins located in the cytoplasm and nucleus, such as Bcl-2, FOXO, IKKα, and MDM2, thereby promoting cell survival, proliferation, growth, and resistance to apoptosis^41^. Bcl-2 family proteins regulate mitochondrial membrane permeability and are significant regulators of mitochondrial apoptosis^42^. This family of proteins consists of two functionally opposite subgroups, the anti-apoptotic members represented by BCL-2 and BCL-XL, and the pro-apoptotic members represented by BAX and BAK^43^. Among them, BCL-2 is the founding member of the family and plays a crucial role in apoptosis. caspase-9 is the initiator and effector of apoptosis and plays an important regulatory role in apoptosis^44^. Akt inhibits the pro-apoptotic members of the Bcl-2 family, such as Bad, Bax, and caspase-9, and upregulates the anti-apoptotic members of Bcl-2 and Bcl -xl and other anti-apoptotic proteins expression, and plays a survival role by regulating the balance between anti-apoptotic proteins (Bcl-2 and Bcl-XL) and pro-apoptotic proteins (Bax)^44,45^. Therefore, we examined the protein expression levels of P-PI3K, P-AKT, BCL-2, and caspase9 by Western blot, and the results showed that the expression of P-PI3K, P-AKT, and BCL-2 was significantly increased in the myocardial tissues of QGQXM-intervened rats, whereas the expression of caspase9 was significantly decreased considerably, which was consistent with the results of a previous study. This suggests that QGQXM can resist apoptosis through the PI3K/AKT signaling pathway, which may be a potential mechanism for its anti-DCM effect. In addition, the results of network pharmacological analysis showed that QGQXM could also resist DCM by regulating HIF-1, MAPK, mTOR, Insulin, Apoptosis, and other signaling pathways, which reflected the advantages of multi-target and multi-pathway regulation of QGQXM.

Apoptosis is a form of programmed cell death that is present in a variety of pathological processes. Numerous studies have demonstrated the presence of apoptosis in the pathogenesis of DCM^46^. A prolonged hyperglycemic environment induces apoptosis in cardiomyocytes, which irreversibly causes a reduction in cardiomyocytes, which in turn reduces myocardial diastolic-contractile function and ultimately leads to cardiac remodeling^47^. Inhibition of apoptosis has therefore become an essential target for DCM therapy. Analysis of the composition of QGQXM showed that various components can exert the effect of inhibiting apoptosis including epimedoside, baicalin, and dulcitin. Icariin, the main active ingredient of the traditional Chinese medicine Epimedium, is a flavonoid that is anti-apoptotic, anti-inflammatory, hypolipidemic, and anti-oxidative stress^48^. Baicalin is one of the main active components of the traditional Chinese medicine Scutellaria baicalensis, which has been shown to alleviate chronic inflammation, lipid metabolism disorders, apoptosis, and oxidative stress^49^. Azaleatin is also a flavonoid with a wide range of pharmacological effects, inhibiting glutamate-induced reactive oxygen species expression, apoptosis, and mitochondrial damage in HT3 cells^26^. Puerarin can reduce CSE-induced apoptosis in human bronchial epithelial cells by modulating the PI3K/Akt/mTOR signaling pathway^50^.

To verify the effect of QGQXM on PI3K-Akt signaling pathway-mediated cardiomyocyte apoptosis, we constructed a DCM rat model and demonstrated the model by cardiac ultrasound, and fasting blood glucose. The experimental results showed that QGQXM could ameliorate cardiomyocyte injury caused by DCM, reduce the degree of myocardial fibrosis, and improve cardiac function. Western blot results showed that the expression of p-PI3K, p-AKT, and Bcl-2 was increased in the QGQXM group. The expression of caspase-9 was decreased, suggesting that QGQXM could activate the PI3K-Akt signaling pathway, increase the expression of Bcl-2, a downstream anti-apoptotic factor, as well as down-regulate the expression of the pro-apoptotic protein caspase-9, exerting an anti-DCM effect by inhibiting cardiomyocyte apoptosis. This is consistent with previous findings.Finally, in our PPI analysis findings, GAPDH emerged as one of the central targets. Recognized routinely as a standard internal reference in western blotting, GAPDH's expression stability under our experimental conditions warranted a deeper investigation. To this end, we conducted the following analysis: First, network pharmacology analysis identified only one component, adenosine, as having a regulatory effect on GAPDH. Adenosine, a traditional Chinese medicine (TCM) component, is present at low concentrations within the complex mixture. Second, the SwissTargetPrediction database indicates only a 0.27 probability of adenosine regulating GAPDH, suggesting low similarity. Due to its concentration and the limitations of predictive models, the actual regulatory effect of adenosine on GAPDH remains unclear. Therefore, our results showed no significant change in GAPDH expression, supporting its continued use as an internal reference protein for western blotting.

The present study also has some limitations. In the mass spectrometry part, we only analyzed the prototypical components in the blood, and further studies are still needed for metabolizing the herbal components after entering the blood. In addition, only in vivo experiments in rats were conducted in this study, and in the future, well-established clinical trials are needed to verify its therapeutic effects.

## Conclusion

QGQXM, as a clinically effective formula, contributes to better heart protection. One of its potential mechanisms involves activating the PI3K-Akt signaling pathway to exert its anti-DCM effects. Additionally, we utilized UPLC-Q/TOF-MS technology in conjunction with network pharmacology to identify key active components. These components may serve as crucial foundations for the efficacy of herbal formulations, such as epimedium glycosides, baicalin, scopolamine, and flavonoids. Therefore, QGQXM, as a potential therapeutic agent for DCM, merits further investigation (Supplementary Material 4).

### Supplementary Information

Supplementary Information 1.

Supplementary Information 2.

Supplementary Information 3.

Supplementary Information 4.

## Data Availability

Raw data for this study can be obtained by contacting the corresponding author. Public data from the following databases: PubChem database (https://pubchem.ncbi.nlm.nih.gov/), SwissTargetPrediction (https://www.swisstargetprediction.ch/), OMIM (http://www.omim.org/), GeneCards database (https://www.genecards.or/).

## Abbreviations

DCM: Diabetic cardiomyopathy
QGQXM: Qigui Qiangxin mixture
UPLC-Q/TOF-MS: Ultra-performance liquid chromatography coupled with quadrupole time of flight mass spectrometry
STZ: Streptozotocin
IDF: International diabetes federation
ACEI: Angiotensin-converting enzyme inhibitor
ARNI: Angiotensin receptor neprilysin inhibitor
βB: Beta-blocker
MRA: Mineralocorticoid receptor antagonists
SGLT2i: Sodium-glucose cotransporter-2 inhibitor
TCM: Traditional Chinese Medicine
PPI: Protein–protein interaction
HE: Hematoxylin–eosin staining
TUNEL: Terminal deoxynucleotidyl transferase-mediated dUTP nick end labeling
PI3K: Phosphatidylinositol 3-kinase
AKT: Serine/threonine kinase
