# Supplementary material for: Exploring the mechanism of diabetic cardiomyopathy treated with Qigui Qiangxin mixture based on UPLC-Q/TOF-MS, network pharmacology and experimental validation
Source: Sci Rep. 2024 May 27;14:12119. doi: 10.1038/s41598-024-63088-7 (PMC11130275; doi:10.1038/s41598-024-63088-7)
Supplement: Supplementary file 1 — Supplementary Information 1. [file 41598_2024_63088_MOESM1_ESM.pdf]

1 Equipment and materials

SCIEX QTOF 6600 time-of-flight mass spectrometer (AB SCIEX, USA); TurboIonSpray ion source (AB SCIEX, USA); Shimadzu Nexera X2 LC-30AD ultra-high-performance liquid chromatograph (Shimadzu); Thermo ST40R low-temperature and high-speed centrifuge (Thermo Fisher Scientific, USA); IKA micro vortex mixer (IKA, Germany). Methanol, acetonitrile and formic acid (Merck, Germany), Milli-Q ultrapure water (Millipore, USA), and the rest of the reagents were analytically pure.

2 Chromatographic conditions

The chromatographic column was ACQUITY UPLC HSS T3 1.8 μm, 2.1 mm× 100 mm (Waters), and the mobile phase was 0.1% formic acid in water (A) - 0.1% formic acid in acetonitrile (B). The gradient elution program was as follows: 0-11 min, 5%~90% B; 11-12 min, 90% B; 12-12.5 min, 90%~5% B. The gradient elution program was as follows; 12.5-15 min, 5% B. Flow rate: 0.3 mL/min; injection plate temperature: 8 ℃; column temperature: 40 ℃; injection volume: 5 μL.

3 Mass spectrometry conditions

The time-of-flight mass spectrometry was performed with TurboIonSpray ion source and ESI positive and negative ion scanning modes. The specific conditions were as follows: Ion Source Gas1 (Gas1): 55, Ion Source Gas2 (Gas2): 55, Curtain gas (CUR): 35, source temperature: 550 ℃, IonSapary Voltage Floating (ISVF): 5500 V/-4500 V (positive and negative). /-IonSapary Voltage Floating (ISVF): 5500 V/4500 V (both positive and negative ion modes); TOF MS scan m/z range: 50-1500 Da, production scan m/z range:

25-1000 Da, TOF MSscan accumulation time 0.25 s/spectra, product ion scan accumulation time 0.035 s/spectra; the secondary mass spectra were obtained by information dependent acquisition (IDA) and high sensitivity mode, Declusteringpotential (DP): ± 60 V (positive and negative ions). Declusteringpotential(DP): ±60 V (both positive and negative ion modes), Collision Energy: 30±15 eV, IDA settings were as follows Excludeisotopes within 4 Da, Candidate ions to monitor per cycle: 15
