# Supplementary material for: Exploring the mechanism of diabetic cardiomyopathy treated with Qigui Qiangxin mixture based on UPLC-Q/TOF-MS, network pharmacology and experimental validation
Source: Sci Rep. 2024 May 27;14:12119. doi: 10.1038/s41598-024-63088-7 (PMC11130275; doi:10.1038/s41598-024-63088-7)
Supplement: Supplementary file 2 — Supplementary Information 2. [file 41598_2024_63088_MOESM2_ESM.pdf]

1. Analytical methods

Aipathwell is a digital pathology image analysis software based on artificial intelligence learning introduced by Servicebio. AI deep learning principle is used to train algorithms based on massive data and integrate them into automated image analysis software. The specific process is as follows:

1 Tracking: automatically locate and delineate the area to be measured along the tissue to be measured, and manually locate according to specific requirements;

2 color selection: according to HSI automatic positive judgment, can be manually corrected according to the specific situation;

3 calculation: according to the demand, the software automatically calculates the positive area in the test area, tissue area and other parameters;

4 Analysis: Gradually calculate the area to be tested at high power. After completion, the analysis results are automatically calculated according to the original basic data and the algorithm formula, and the report is generated.

2. Evaluation item

Masson positive area ratio = Positive area/tissue area [1]

3. References

[1] Impact of myocardial fibrosis on left ventricular remodelling, recovery, and outcome after transcatheter aortic valve implantation indifferent haemodynamic subtypes of severe aortic stenosis[J]. Miriam Puls, 2020, 41:1903-1914. IF:22.673.
