# Supplementary material for: Exploring the mechanism of diabetic cardiomyopathy treated with Qigui Qiangxin mixture based on UPLC-Q/TOF-MS, network pharmacology and experimental validation
Source: Sci Rep. 2024 May 27;14:12119. doi: 10.1038/s41598-024-63088-7 (PMC11130275; doi:10.1038/s41598-024-63088-7)
Supplement: Supplementary file 3 — Supplementary Information 3. [file 41598_2024_63088_MOESM3_ESM.pdf]

1、Analytical method

Aipathwell® is a digital pathology image analysis software based on artificial intelligence learning introduced by Servicebio®. AI deep learning principle is used to train algorithms based on massive data and integrate them into automated image analysis software. The specific process is as follows:

1, tracking: automatically locate the tissue and delineate the area to be measured along the tissue to be measured, can be manually positioned according to specific requirements.

2, color selection: According to HSI (Hue, Saturation, Intensity) automatically positive judgment and positive grade: weak positive light yellow, calculated as 1 point; Moderate positive brown-yellow color, 2 points; Strong positive tan, 3 points. It can be manually corrected according to the specific situation.

3, calculation: according to the demand, the software automatically locates the nucleus and expands the cytoplasm range; The number and area of weak, medium and strong positive cells were calculated. integrated optical density (IOD); Tissue area and other different parameters.

4. Analysis: Gradually calculate the area to be tested at high power. After completion, each project is calculated automatically according to the original basic data and the algorithm formula to obtain the analysis results, and generate a report.

2, evaluation items (according to the analysis needs can selectively analyze the number of positive cells or positive area)

Analysis of correlation parameters of positive cell number

1. Positive cell ratio = Number of positive cells/total number of cells [1] [2] [6]. Reflecting the number of positive cells of the same type, it is suitable for the case of a single cell type. For example, the ratio of proliferation-related ki67, PCNA and other positive tumor cells to the total number of tumor cells in tumor tissue.

2. H-score: Histochemistry score (Histochemistry score) is a histological scoring method to deal with immunohistochemistry, the proportion of positive cells in each section and its staining intensity into the corresponding value. To achieve the purpose of comprehensive semi-quantitative analysis of the positive depth and positive number of tissue immunostaining [4][5][6][7][8]. H-Score (Σ (pi×i) = (percentage of weak intensity cells

×1)+(percentage of moderate intensity cells ×2)+(percentage of strong intensity cells ×3), where i indicates the grade classification of positive cells: negative without staining, 0 points; Weak positive light yellow, count 1 point; Moderate positive brown-yellow color, 2 points; Strong positive tan is 3 points. pi represents the percentage of positive cells of the corresponding grade). H-score is a value between 0 and 300, and the larger the value is, the stronger the comprehensive positive strength is in terms of the depth of positive and the number of positive. The most widely applicable.

3. IRS=SI (positive intensity) ×PP (positive cell ratio) [4]. SI can be divided into 3 grades, grade 0 no positive staining, grade 1 light yellow weak positive, grade 2 brown moderate positive, grade 3 brown strong positive. PP can be divided into 4 levels, 0 level is 0 ~ 5%, 1 level is 6% ~ 25%, 2 level is 26% ~ 50%, 3 level is 51% ~ 75%, 4 level is >75%. The larger the IRS data, the stronger the comprehensive positive intensity of the two aspects of positive depth and positive number, which is common in the positive intensity analysis of tumor tissue.

4. Positive cell density = number of positive cells/tissue area to be measured [1]. Reflects the number of positive cells per unit area, and is mostly used to evaluate the distribution and number of a certain type of cells in the tissue. For example, the distribution and number of CD3, CD4, CD8 and other positive lymphocytes in the tumor microenvironment.

5. Average optical density = cumulative optical density value IOD/ positive pixel area [5]. Reflect the average depth of positive signal, use positive depth to evaluate the positive strength. It is suitable for the situation where positive tablets are widely expressed.

3. Analysis of relevant parameters of positive area

1. H-score: Histochemistry score (Histochemistry score) is a histological scoring method to deal with immunohistochemistry, the ratio of positive area in each section and its staining intensity into the corresponding value. To achieve the purpose of comprehensive semi-quantitative analysis of the positive depth and positive number of tissue immunostaining [4][5][6][7][8] [9]. H-Score (Σ (pi × i) = (percentage of weak intensity cells × 1)+(percentage of moderate intensity cells ×2)+(percentage of strong intensity cells ×3), where i indicates the grade classification of positive cells: negative without staining, 0 points; Weak positive light yellow, count 1 point; Moderate positive brown-yellow color, 2 points; Strong positive tan is 3 points. pi represents the percentage of positive cells of the

corresponding grade). H-score is a value between 0 and 300, and the larger the value is, the stronger the comprehensive positive strength is in terms of the depth of positive and the number of positive. The most widely applicable.

2. Average optical density =IOD/ positive pixel area [5]. Reflect the average depth of positive signal, use positive depth to evaluate the positive strength. It is suitable for the situation where positive tablets are widely expressed.

3. Positive area ratio = positive area/tissue area [3]. Reflect the number of positive area, suitable for positive tablets and widespread expression of the situation. For example: analysis of the extent of Aβ-positive amyloid deposition in AD model brain tissue.

4. Positive surface density =IOD/ tissue pixel area [5] [6]. It reflects the average depth of positivity in the tissue area to be measured, and is suitable for the situation where the positivity is expressed in slices and distributed unevenly on the tissue.

4、References

[1] Benonisson Hreinn, Altıntaş Işıl, Sluijter Marjolein, Verploegen Sandra, Labrijn Aran F, Schuurhuis Danita H, Houtkamp Mischa A, Verbeek J Sjef, Schuurman Janine, van Hall Thorbald. CD3-Bispecific Antibody Therapy Turns Solid Tumors into Inflammatory Sites but Does Not Install Protective Memory [J]. Molecular cancer therapeutics, 2019, 18(2). IF：5.04

[2] Yawei X, Hongxia C, Zixiang G, et al. Circular RNA circ0007360 Attenuates Gastric Cancer Progression by Altering the miR-762/IRF7 Axis [J]. Frontiers in Cell and Developmental Biology, 2022, 10. IF：7.4

[3] LingGe Z, ZhangHua W, ShaSha L, et al. The Potential Roles of CHI3L1 in Failed Autologous Arteriovenous Fistula in End-Stage Renal Disease [J]. The Tohoku journal of experimental medicine, 2023, advpub(0). IF：5.2

[4] Yin JZ, Shi XQ, Wang MD, Du H, Zhao XW, Li B, Yang MH. Arsenic trioxide elicits anti-tumor activity by inhibiting polarization of M2-like tumor-associated macrophages via Notch signaling pathway in lung adenocarcinoma. Int Immunopharmacol. 2023 Apr;117:109899. doi: 10.1016/j.intimp.2023.109899. Epub 2023 Feb 22. PMID: 36827926. IF：7.2

[5] Zhao R, Zhou Y, Shi H, Ye W, Lyu Y, Wen Z, Li R, Xu Y. Effect of Gestational Diabetes on Postpartum Depression-like Behavior in Rats and Its Mechanism. Nutrients. 2022 Mar

14; 14 (6):1229. doi: 10.3390/nu14061229. PMID: 35334886; PMCID: PMC8953401. IF：7.7

[6] Wang H, Chiang C, Xue C, Zhou L, Li S, Zhou Y, Zhang Z, Xie M, Xiao T, Hu H, Zhu L, Long C, Zou Y, Wang T, Zheng D. Dezocine induces apoptosis in human cervical carcinoma Hela cells via the endoplasmic reticulum stress pathway. Toxicol Res (Camb). 2022 May 30; 11(3):498-510. doi: 10.1093/toxres/tfac026. Erratum in: Toxicol Res (Camb). 2022 Dec 01;12(1):143. PMID: 35782639; PMCID: PMC9244724. IF：5.3

[7] Cheng M, Xu J, Ding K, Zhang J, Lu W, Liu J, Gao J, Alugupalli KR, Liu H. Attenuation of relapsing fever neuroborreliosis in mice by IL-17A blockade. Proc Natl Acad Sci U S A. 2022 Oct 18;119(42):e2205460119. doi: 10.1073/pnas.2205460119. Epub 2022 Oct 10. PMID: 36215473; PMCID: PMC9586318. IF：11.1

[8] Zheng N, Wen R, Zhou L, Meng Q, Zheng K, Li Z, Cao F, Zhang W. Multiregion single cell analysis reveals a novel subtype of cancer-associated fibroblasts located in the hypoxic tumor microenvironment in colorectal cancer. Transl Oncol. 2023 Jan; 27:101570. doi: 10.1016/j.tranon.2022.101570. Epub 2022 Nov 10. PMID: 36371957; PMCID: PMC9660844. IF：6.2

[9] Lu T, Zhang Z, Zhang J, Pan X, Zhu X, Wang X, Li Z, Ruan M, Li H, Chen W, Yan M. CD73 in small extracellular vesicles derived from HNSCC defines tumour-associated immunosuppression mediated by macrophages in the microenvironment. J Extracell Vesicles. 2022 May; 11(5): e12218. doi: 10.1002/jev2.12218. PMID: 35524455; PMCID: PMC9077142. IF：15.9
