# Supplementary figures and images for: Exploring the mechanism of diabetic cardiomyopathy treated with Qigui Qiangxin mixture based on UPLC-Q/TOF-MS, network pharmacology and experimental validation
Source: Sci Rep. 2024 May 27;14:12119. doi: 10.1038/s41598-024-63088-7 (PMC11130275; doi:10.1038/s41598-024-63088-7)

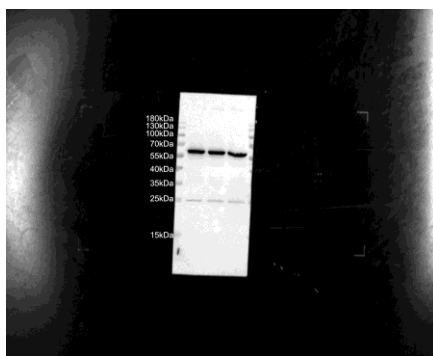

1 AKT

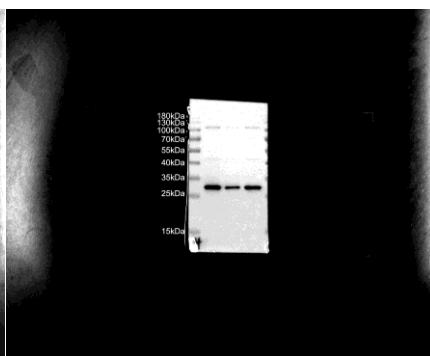

1 BCL-2

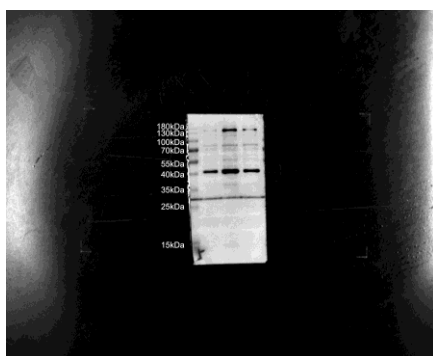

1 CAS-9

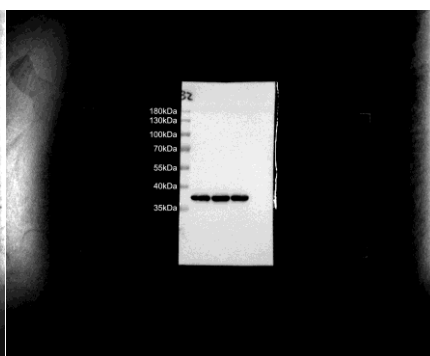

1 GAPDH

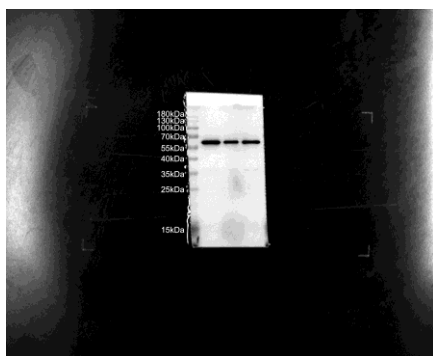

1 P-AKT

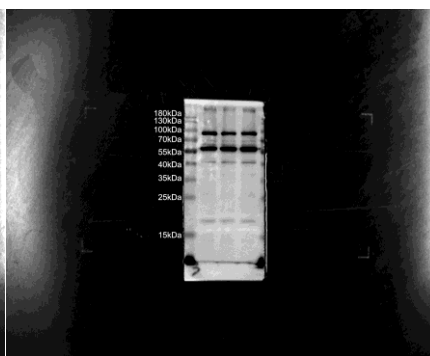

1 PI3K

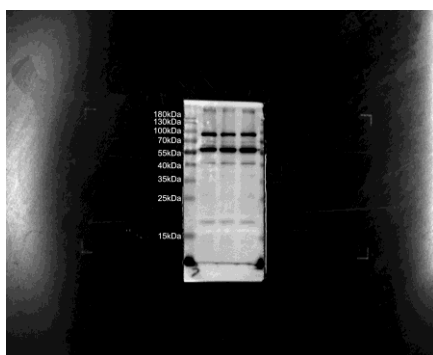

1 P-PI3K

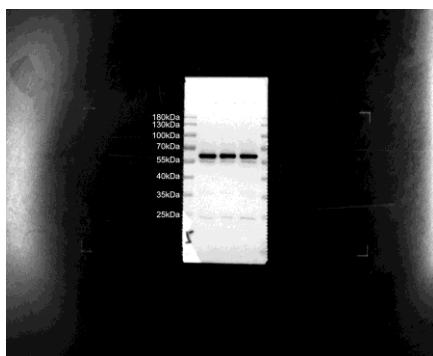

2 AKT

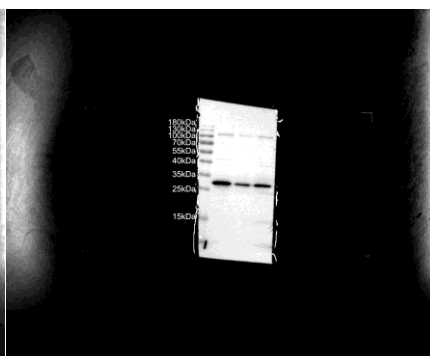

2 BCL-2

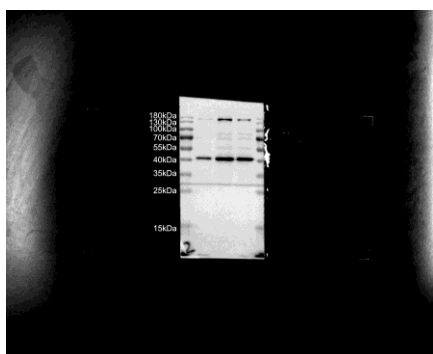

2 CAS-9

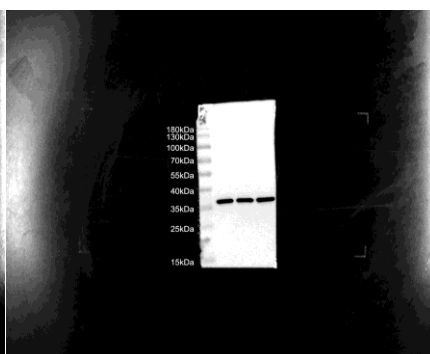

2 GAPDH

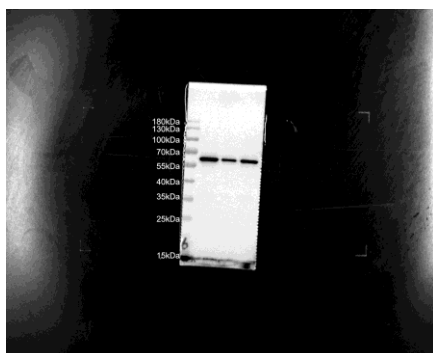

2 P-AKT

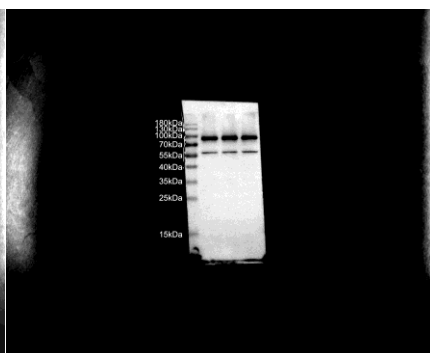

2 PI3K

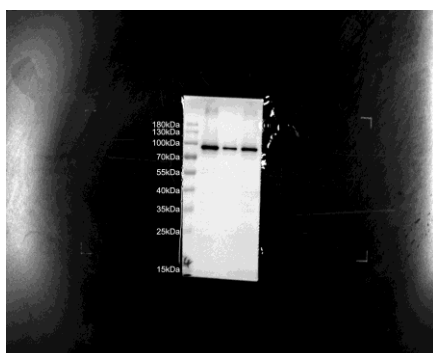

2 P-PI3K

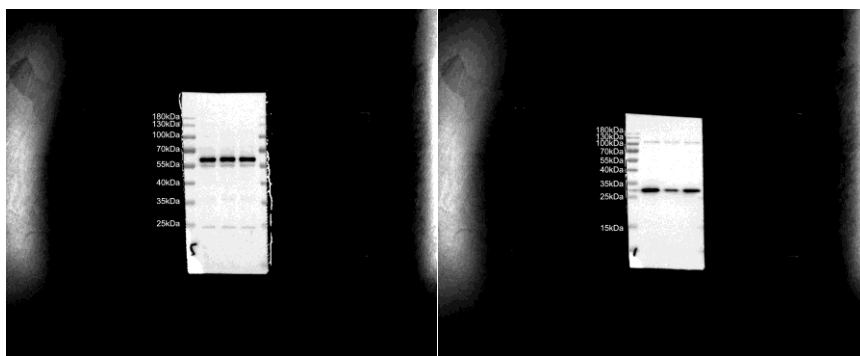

3 AKT

3 BCL-2

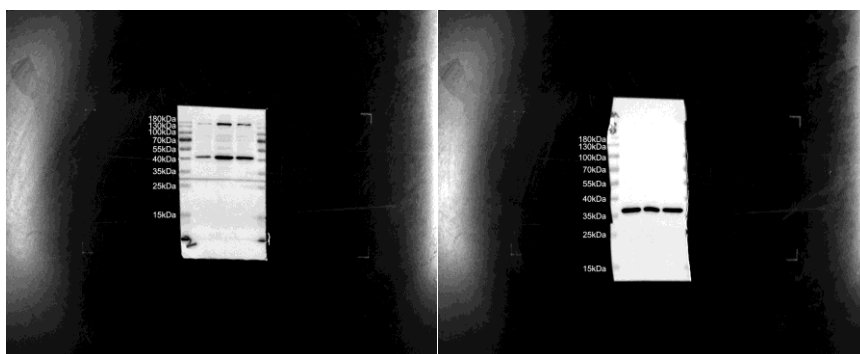

3 CAS-9

3 GAPDH

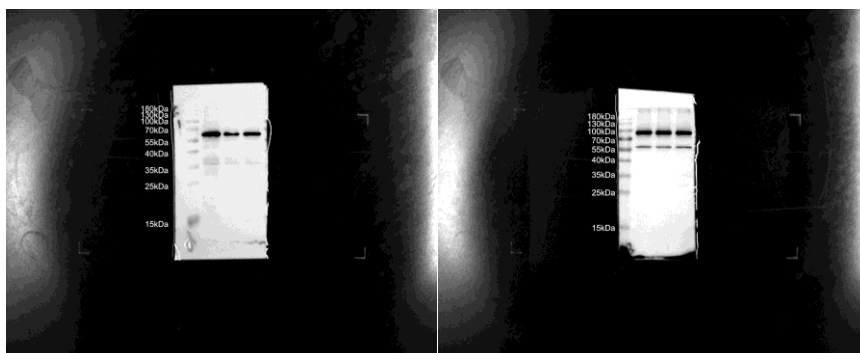

3 P-AKT

3 PI3K

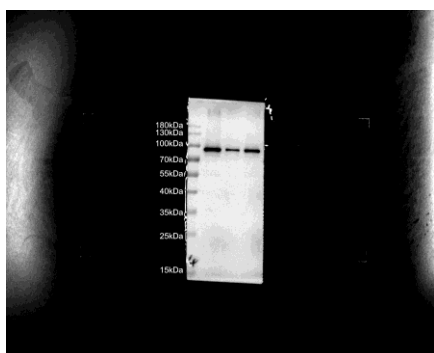

3 P-PI3K

Supplement: Supplementary file 4 — Supplementary Information 4. [file 41598_2024_63088_MOESM4_ESM.pdf]
